# Risk of ischemic stroke associated with anti-rheumatic agents in patients with rheumatoid arthritis: A nationwide population-based case-control study

**DOI:** 10.1371/journal.pone.0326311

**Published:** 2025-06-17

**Authors:** Soo Min Ahn, Seonok Kim, Ye-Jee Kim, Seokchan Hong, Chang-Keun Lee, Bin Yoo, Ji Seon Oh, Yong-Gil Kim

**Affiliations:** 1 Division of Rheumatology, Department of Internal Medicine, Asan Medical Center, University of Ulsan College of Medicine, Seoul, Republic of Korea; 2 Department of Clinical Epidemiology and Biostatistics, Asan Medical Center, University of Ulsan College of Medicine, Seoul, Republic of Korea; 3 Department of Information Medicine, Big Data Research Center, Asan Medical Center, Seoul, Republic of Korea; National Hospital Organization Kumamoto Saishun Medical Center, JAPAN

## Abstract

**Introduction:**

Patients with rheumatoid arthritis (RA) face a significantly higher risk of major adverse cardiovascular events, including stroke, due to the pivotal role of inflammation in atherosclerosis and thrombosis. Various anti-rheumatic agents may influence stroke risk either by increasing or decreasing it, and this effect remains unclear. This study aimed to investigate the association between different anti-rheumatic agents and the risk of incident stroke in patients with RA using a nationwide claims database.

**Methods:**

In this nested case-control study, the Korean Health Insurance Review and Assessment data of 35,133 patients newly diagnosed with seropositive RA from January 2011 to December 2020 were used. Incident ischemic stroke cases were identified and matched with randomly selected controls at a 1:4 ratio. The usage of anti-rheumatic agents was measured from the date of RA diagnosis to the index date and stratified in terms of exposure time and duration. The risk of stroke associated with each anti-rheumatic agent was estimated using conditional logistic regression and adjusted for comorbidities and concomitant drug use.

**Results:**

Of the 35,133 patients, 1,386 (3.9%) cases were newly diagnosed with new-onset stroke. Thus, 1,384 stroke cases and 5,499 controls with newly diagnosed RA were included in the analysis. Current exposure to sulfasalazine (aOR: 0.79, 95% CI: 0.65–0.97) and hydroxychloroquine (aOR: 0.83, 95% CI: 0.72–0.96) was associated with a decreased risk of stroke, while current exposure to glucocorticoids (aOR: 1.71, 95% CI: 1.46–2.00) and tocilizumab (aOR: 3.47, 95% CI: 1.70–7.08) was related to an increased risk of stroke.

**Conclusion:**

In this nationwide cohort study of patients with RA, treatment with sulfasalazine and hydroxychloroquine was associated with a decreased risk of stroke, while glucocorticoids and tocilizumab were linked to an increased risk of stroke. The association of tocilizumab with stroke should be cautiously interpreted due to the statistical limitations.

## Introduction

Rheumatoid arthritis (RA) is a chronic, systemic, immune-mediated inflammatory disorder primarily affecting the joints and is often accompanied by extra-articular manifestations [1]. Patients with RA have a higher risk of major adverse cardiovascular events (MACE), including stroke, than the general population [2,3]. Notably, these events represent the leading causes of death among patients with RA [4]. Inflammation plays a pivotal role in stroke development, contributing to atherosclerosis and blood clot formation, which significantly increases the risk of stroke [5]. Anti-rheumatic agents with anti-inflammatory properties influence various processes involved in the development and progression of atherosclerosis, including glucose regulation, blood pressure modulation, cholesterol metabolism, thrombosis, and coagulation [6]. Non-steroidal anti-inflammatory drugs (NSAIDs) and steroids increase cardiovascular risk, whereas anti-rheumatic agents, such as conventional synthetic disease-modifying anti-rheumatic drugs (csDMARDs) and biological disease-modifying anti-rheumatic drugs (bDMARDs), are likely to lower cardiovascular risk [6]. However, factors such as race, diet, climate, and socioeconomic conditions can influence cardiovascular risk. Therefore, the effect of each anti-rheumatic agent on cardiovascular diseases, especially stroke, remains controversial.

Studies comparing different drugs used for RA treatment have provided insufficient evidence to draw definitive conclusions about cardiovascular risk, particularly the risk of stroke associated with various treatment regimens. Therefore, this study aimed to examine the association between the use of different anti-rheumatic agents and the risk of ischemic stroke in patients who had RA and did not previously have a stroke by using a nationally representative cohort of medical claims data in Korea.

## Materials and methods

### Data source

In this retrospective population-based nationwide study, data from the Korean Health Insurance Review and Assessment Service (HIRA) claims database were used. This database contains health-related information on approximately 50 million South Koreans covered under the National Health Insurance (NHI) program [7]. The HIRA database contains information on patient demographics (e.g., 5-year age intervals, sex), diagnoses, medical and surgical procedures, and prescriptions [8].

This study was in compliance with the ethical guidelines of the Declaration of Helsinki. Because of the retrospective nature of the study, the requirement for informed consent was waived by the Institutional Review Board of Asan Medical Center (approval no. 2021−1365). The data were accessed for research purposes from August 12, 2022, to July 6, 2023. The de-identified data can be accessed upon approval of Health Insurance Review and Assessment service, Korea. The authors did not have access to any information that could identify individual participants during or after data collection.

### Study population

The diagnosis of RA was determined using the International Classification of Diseases, 10th Revision (ICD-10), as adapted to the Korean healthcare system, along with the Korean Rare Intractable Disease (RID) registration code for RA. In the RID registration program, qualified physicians diagnose rare diseases, including some rheumatic diseases, according to the unified diagnostic criteria distributed by the NHI. Diagnoses are registered following a review conducted by the relevant healthcare institution and the NHI. In the Korean RID system, RA is diagnosed according to the 2010 American College of Rheumatology/European League Against Rheumatology classification criteria for RA and a positive test for rheumatoid factor or anti-citrullinated peptide antibodies [9].

Patients with newly diagnosed seropositive RA (ICD-10 code M05; RID code V223) between January 1, 2011 and December 31, 2020 were included in the RA cohort. Newly diagnosed RA was defined as patients receiving the RA diagnostic code for the first time and having no prior record of anti-rheumatic drug prescriptions from January 2010 until the date of RA code assignment. The following patients were selected to increase the validity of the diagnosis: those who had visited an outpatient clinic at least twice, received a prescription for anti-rheumatic agents, and were registered in the Korean RID system. Patients with a history of stroke or acute myocardial infarction (AMI) before RA diagnosis were excluded. The observation period for risk assessment was set from a minimum of 1 year to a maximum of 10.2 years. Only individuals aged 20 years or older were included. The following exclusion criteria were applied to minimize potential confounding effects on the evaluation of the influence of RA treatment: seronegative RA, other rheumatic diseases (e.g., systemic lupus erythematosus, Sjogren’s syndrome, mixed connective tissue disease, and inflammatory myositis), interstitial lung disease, solid organ transplantation, cancer, human immunodeficiency virus infection, and/or end-stage renal disease requiring dialysis (S1 Table). Individuals with an observation period shorter than 90 days were excluded.

### Cases and controls

In this study, “cases” referred to individuals with RA who were subsequently diagnosed with an ischemic stroke. Specifically, they were identified as patients who were hospitalized with a newly recorded diagnosis of acute ischemic stroke (ICD-10 code I63) and with stroke-related images (S2 Table). Incident stroke cases were matched to controls from the existing RA cohort at a 1:4 ratio using a greedy algorithm to establish a control group. The matching criteria were age, sex, and the year of RA diagnosis. The index date was defined as the date of the first recorded stroke diagnosis; for controls, the index date was the same as that of their matched case.

### Assessment of exposure to anti-rheumatic agents

Exposure to anti-rheumatic agents was determined on the basis of prescription records. Anti-rheumatic agents included csDMARDs (i.e., hydroxychloroquine, methotrexate, leflunomide, sulfasalazine, and tacrolimus), bDMARDs (i.e., anti-tumor necrosis factor-alpha [TNF-α] agents [i.e., adalimumab, etanercept, golimumab, and infliximab], abatacept, and tocilizumab), Janus kinase (JAK) inhibitors (i.e., tofacitinib, baricitinib, and upadacitinib), and glucocorticoids.

Drug exposure was evaluated from the time of RA diagnosis to the occurrence of stroke, with data for individuals diagnosed in 2011 being retrospectively collected back to 2010. Groups were classified based on the duration and exposure risk window for each anti-rheumatic agent. First, the exposure risk window was further categorized into two: current exposure (prescription within 3 months of the index date) and past exposure (prescription ended more than 3 months before the index date) groups based on the timing of anti-rheumatic agent exposure before stroke occurred. Next, based on a 12-month timeframe, exposure was divided into short- (<12 months) and long-term (≥12 months) groups. Lastly, patients were categorized into five groups: non-exposure, past short-term exposure, past long-term exposure, current short-term exposure, and current long-term exposure. Patients in the non-exposed group might still have been treated with other DMARDs although they were not exposed to the primary drug under investigation.

### Confounding variables

All potential covariates influencing stroke risk were evaluated using data from the year preceding the index date. Data on age groups at RA diagnosis, sex, comorbidities (hypertension, dyslipidemia, diabetes mellitus, carotid stenosis, asthma, chronic obstructive pulmonary disease, chronic kidney disease, congestive heart failure, and atrial fibrillation), and medication use (low-dose aspirin, NSAIDs, statins, and anti-hypertensive drugs) were collected for each patient. The Charlson Comorbidity Index (CCI) was calculated to assess underlying comorbidities [10].

### Statistical analysis

Participant characteristics, including age groups, comorbidities, and medications, were thoroughly analyzed. Categorical variables were presented as numbers with percentages, while continuous variables were expressed as means with standard deviations. The balance in the distribution of baseline characteristics between stroke patients and matched controls was quantified using the standardized mean difference. Conditional logistic regression models were used to assess the specific impact of each anti-rheumatic agent on the risk of stroke in comparison with non-exposure to the respective agents. The risk of new-onset stroke was further evaluated after adjusting for comorbidities, CCI, and the use of NSAIDs, low-dose aspirin, statins, antihypertensive agents, and other anti-rheumatic agents. Results were reported using adjusted odds ratios (aORs) and 95% confidence intervals (CIs). Data with *p* value <0.05 were considered statistically significant. All statistical analyses were conducted using SAS Enterprise Guide software (version 7.1; SAS Institute, Cary, NC, USA).

## Results

### Study population and baseline characteristics

**Fig 1:**
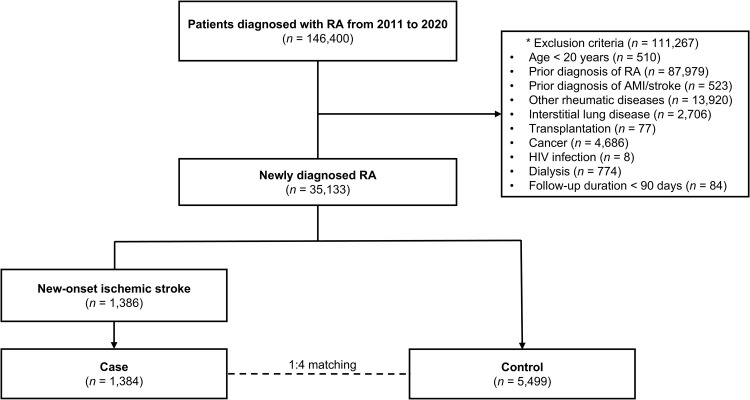
Study population selection. Abbreviations: RA, rheumatoid arthritis; AMI, acute myocardial infarction; HIV, human immunodeficiency virus. *Other rheumatic diseases included systemic lupus erythematosus, mixed connective tissue diseases, Sjogren’s syndrome, and inflammatory myositis.

In the Korean HIRA claims database, 35,133 patients were newly diagnosed with seropositive RA between 2011 and 2020, and they met the inclusion and exclusion criteria. Among these patients, 1,386 (3.9%) were diagnosed with new-onset stroke during a follow-up period of up to 10.2 years (median: 9.92 years, interquartile range: 9.78–10.00 years). After excluding two unmatched cases, 1,384 stroke patients were matched 1:4–5,499 non-stroke patients based on sex, age, and year of RA diagnosis using a nested case-control design (Fig 1).

The baseline characteristics of the study individuals are shown in Table 1. The majority of patients in both groups were between 60 and 69 years of age, and 79% were women. The prevalence of hypertension, dyslipidemia, diabetes mellitus, asthma, chronic obstructive pulmonary disease, chronic kidney disease, congestive heart failure, and atrial fibrillation of patients who had a stroke was higher than that of patients who did not have a stroke. The proportion of patients who had a stroke with high CCI scores was significantly higher than that of patients who did not have a stroke.

**Table 1:** Baseline characteristics of cases and controls.

Characteristics	Case (n = 1384)	Control (n = 5499)	Total (n = 6883)	*P*-value
**Female sex**	1090 (78.8)	4347 (79.1)	5437 (79.0)	0.839
**Age group**, years				
20–29	1 (0.1)	4 (0.1)	5 (0.1)	>0.999
30–39	6 (0.4)	24 (0.4)	30 (0.4)	
40–49	59 (4.3)	236 (4.3)	295 (4.3)	
50–59	219 (15.8)	875 (15.9)	1094 (15.9)	
60–69	533 (38.5)	2131 (38.8)	2664 (38.7)	
70–79	487 (35.2)	1920 (34.9)	2407 (35.0)	
>80	79 (5.7)	309 (5.6)	388 (5.6)	
**Co-morbidities**				
Hypertension	993 (71.7)	2816 (51.2)	3809 (55.3)	<0.001
Dyslipidemia	745 (53.8)	2415 (43.9)	3160 (45.9)	<0.001
Diabetes mellitus	430 (31.1)	1056 (19.2)	1486 (21.6)	<0.001
Carotid stenosis	19 (1.4)	40 (0.7)	59 (0.9)	0.030
Asthma	183 (13.2)	570 (10.4)	753 (10.9)	0.003
Chronic obstructive pulmonary disease	58 (4.2)	148 (2.7)	206 (3.0)	0.005
Chronic kidney disease	64 (4.6)	117 (2.1)	181 (2.6)	<0.001
Congestive heart failure	171 (12.4)	270 (4.9)	441 (6.4)	<0.001
Atrial fibrillation	83 (6.0)	87 (1.6)	170 (2.5)	<0.001
**Charlson Comorbidity Index**				
0	268 (19.4)	2130 (38.7)	2398 (34.8)	<0.001
1–2	635 (45.9)	2456 (44.7)	3091 (44.9)	
3–4	325 (23.5)	721 (13.1)	1046 (15.2)	
>5	156 (11.3)	192 (3.5)	348 (5.1)	
**Co-medications**				
NSAIDs	969 (70.0)	3303 (60.1)	4272 (62.1)	<0.001
Low-dose aspirin	232 (16.8)	440 (8.0)	672 (9.8)	<0.001
Statins	422 (30.5)	1322 (24.0)	1744 (25.3)	<0.001
Anti-hypertensive agents	969 (70.0)	2770 (50.4)	3739 (54.3)	<0.001

Values are presented as number (%). Abbreviations: NSAID, non-steroidal anti-inflammatory drugs.

Table 2 presents the association between current exposure to each anti-rheumatic agent and ischemic stroke, with non-exposure and past exposure to each anti-rheumatic agent as the reference. The stroke risk was higher in groups with current exposure to glucocorticoids (aOR = 1.71, 95% CI = 1.46–2.00) and tocilizumab (aOR = 3.47, 95% CI = 1.70–7.08) compared to the non-exposure and past exposure groups. Conversely, the risks of stroke of the groups with current exposure to hydroxychloroquine (aOR = 0.83, 95% CI = 0.72–0.96) and sulfasalazine (aOR = 0.79, 95% CI = 0.65–0.97) were lower than those of the non-exposure and past exposure groups.

**Table 2:** Association between current exposure to anti-rheumatic agents and ischemic stroke, with non-exposure and past exposure to each anti-rheumatic agent as reference.

Medication	Case (n = 1,384)	Control (n = 5,499)	Matched OR (95% CI)	Adjusted OR^*^ (95% CI)
Hydroxychloroquine	433 (31.3)	1799 (32.7)	0.93 (0.82–1.06)	0.83 (0.72–0.96)
Methotrexate	795 (57.4)	3042 (55.3)	1.10 (0.97–1.24)	0.95 (0.83–1.09)
Leﬂunomide	436 (31.5)	1315 (23.9)	1.51 (1.32–1.72)	1.11 (0.95–1.29)
Sulfasalazine	159 (11.5)	749 (13.6)	0.82 (0.68–0.98)	0.79 (0.65–0.97)
Tacrolimus	109 (7.9)	339 (6.2)	1.31 (1.04–1.64)	1.12 (0.88–1.43)
Glucocorticoids	1055 (76.2)	3308 (60.2)	2.19 (1.91–2.52)	1.71 (1.46–2.00)
Anti-TNF-α agents	35 (2.5)	173 (3.1)	0.79 (0.55–1.15)	0.72 (0.48–1.06)
Abatacept	6 (0.4)	26 (0.5)	0.91 (0.38–2.22)	1.01 (0.39–2.56)
Tocilizumab	16 (1.2)	21 (0.4)	3.02 (1.58–5.80)	3.47 (1.70–7.08)
Janus kinase inhibitors	7 (0.5)	13 (0.2)	2.21 (0.87–5.64)	2.29 (0.82–6.45)

Abbreviations: OR, odds ratio; CI, confidence interval; TNF-α, tumor necrosis factor-alpha.

* adjusted for hypertension, dyslipidemia, diabetes mellitus, carotid stenosis, asthma, chronic obstructive pulmonary disease, chronic kidney disease, congestive heart failure, atrial fibrillation, Charlson comorbidity index (0, 1–2, 3–4, 5+), and use of non-steroidal anti-inflammatory drugs, low-dose aspirin, statins, anti-hypertensive agents, and other anti-rheumatic agents.

**Fig 2:**
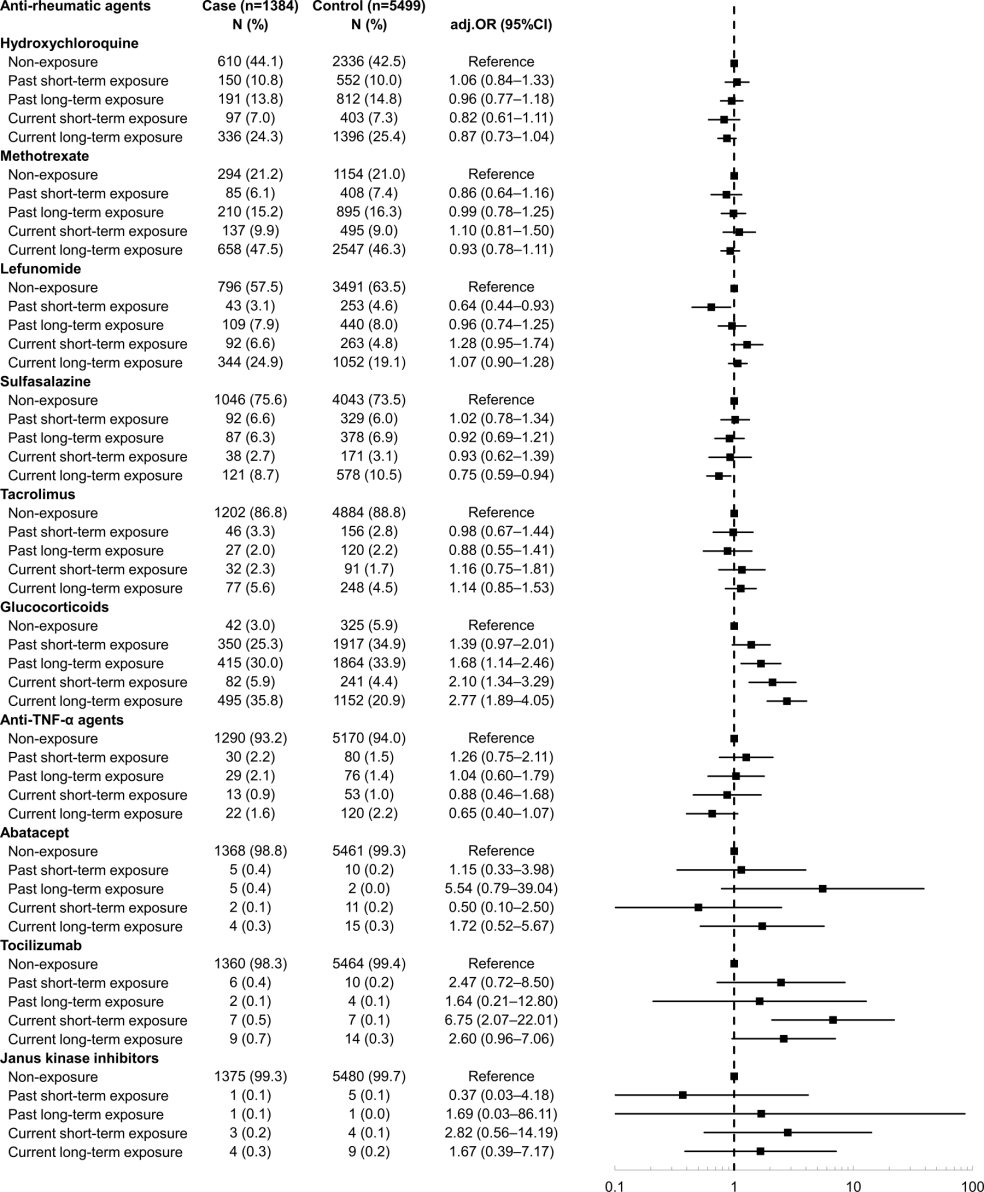
Risk of ischemic stroke according to the use of each anti-rheumatic agents. Abbreviations: adj.OR, adjusted odds ratio; CI, confidence interval; TNF-α, tumor necrosis factor-alpha.

Fig 2 shows the risk of stroke according to the duration and exposure time of each anti-rheumatic agent. The risks of stroke of the groups with glucocorticoid exposure were higher than those of the non-exposure group (past long-term exposure: aOR = 1.68, 95% CI = 1.14–2.46; current short-term exposure: aOR = 2.10, 95% CI = 1.34–3.29; current long-term exposure: aOR = 2.77, 95% CI = 1.89–4.05). The risk of stroke of the group with current short-term exposure to tocilizumab was higher than that of the non-exposure group (aOR: 6.75, 95% CI: 2.07–22.01). Conversely, the risks of stroke of the groups with current long-term exposure to sulfasalazine (aOR: 0.75, 95% CI: 0.59–0.94) or past short-term exposure to leflunomide (aOR: 0.64, 95% CI: 0.44–0.93) were lower than those of the non-exposure group.

Adjusted for hypertension, dyslipidemia, diabetes mellitus, carotid stenosis, asthma, chronic obstructive pulmonary disease, chronic kidney disease, congestive heart failure, atrial fibrillation, Charlson comorbidity index (0, 1–2, 3–4, 5+), and use of non-steroidal anti-inflammatory drugs, low-dose aspirin, statins, anti-hypertensive agents, and other anti-rheumatic agents

S3 Table presents the proportions of patients exposed to each anti-rheumatic agent at any time during the observation period and those with current exposure (within 3 months of the index date). As shown in S3 Table, methotrexate and hydroxychloroquine were the most commonly prescribed medications, whereas biologics and JAK inhibitors were less frequently used.

## Discussion

This population-based nested case-control study revealed that current exposure to glucocorticoids and tocilizumab were associated with an increased risk of stroke, whereas current exposure to sulfasalazine and hydroxychloroquine was associated with a decreased risk of stroke. Specifically, past long-term and current exposure to glucocorticoids and current short-term exposure to tocilizumab were linked to an increased risk of stroke. Conversely, current long-term exposure to sulfasalazine was associated with a decreased risk of stroke. In the present study, 3.9% of newly diagnosed patients with RA in Korea developed acute ischemic stroke. Consistently, a similar study that used claims data in Taiwan reported that 5.4% of patients with RA developed ischemic stroke [11].

Glucocorticoids not only have anti-inflammatory effects but also adverse effects on glucose metabolism, blood pressure, and cholesterol metabolism, which can influence the development of atherosclerosis [6]. Their use increases the risk of cardiovascular disease in patients with RA in a dose- and duration-dependent manner [12,13]. The present study demonstrated that exposure to glucocorticoids was related to an increased risk of stroke in patients with RA. Specifically, the risk of stroke increased incrementally with glucocorticoid exposure, and the risk posed by current exposure was higher than that by past exposure. The risk of ischemic stroke of those with current prolonged exposure to glucocorticoids was increasingly higher than that with short-term exposure. These results emphasized the importance of considering the potential risk of ischemic stroke associated with glucocorticoid use in patients with RA. Therefore, healthcare practitioners should thoughtfully consider these aspects to minimize steroid usage when possible. Further research should identify the factors that significantly influence stroke occurrence, such as the effect of glucocorticoids on the risk of cardiovascular disease compared with RA disease activity that may be inadequately controlled and necessitate glucocorticoid exposure.

Tocilizumab, a humanized monoclonal antibody that targets both the soluble and membranous forms of interleukin-6 (IL-6) receptor, exacerbates dyslipidemia by blocking IL-6 signaling and inducing apolipoprotein expression in the liver [14]. However, recent studies have shown that it does not significantly increase the risk of cardiovascular disease. In a randomized controlled study of patients with RA treated with etanercept (n = 1,542) versus those treated with tocilizumab (n = 1,538), tocilizumab was not inferior in terms of the risk of stroke during a mean follow-up of 3.2 years, with a hazard ratio (HR) of 1.53 (95% CI: 0.80–2.92) [15]. Additionally, an analysis of three large US healthcare claims databases showed no evidence of an increased cardiovascular risk among patients who had RA and switched from bDMARDs or JAK inhibitors to tocilizumab compared with those who switched to an anti-TNF-α agent [16]. However, our study found that current exposure to tocilizumab was significantly associated with an increased risk of stroke, particularly in the group with current short-term exposure to tocilizumab. Despite the relatively small number of tocilizumab users, this association reached statistical significance with a notably high aOR of 3.47 (95% CI: 1.70–7.08), suggesting a potentially important signal. Nevertheless, the reverse exposure–time relationship (i.e., lower risk with longer exposure) does not strongly support a direct causal relationship. A previous case report described ischemic stroke caused by reversible cerebral vasoconstriction at 3 months after tocilizumab administration in a patient with RA [17]; however, this rare case alone cannot explain the magnitude of risk observed in our population-level analysis. During the acute phase of stroke, the inflammatory cytokine IL-6 increases substantially, worsening ischemic damage. Conversely, during the recovery phase of stroke, IL-6 contributes to neuroprotection and facilitates ischemic brain tissue restoration [18]. After IL-6 signaling inhibition, the potential for an increased risk or exacerbation of stroke cannot be conclusively ruled out because the intricate role of IL-6 in the context of stroke is multifaceted. In the general population and among patients with RA, serum IL-6 and C-reactive protein (CRP) are considered predictive of a cardiovascular risk [19,20]. In real-world practice, tocilizumab may be preferred for patients with increased CRP levels; however, this preference could introduce a channeling bias, resulting in certain characteristics that make patients more likely to receive specific treatments. Additionally, because tocilizumab considerably suppresses acute-phase reactants in RA, the disease activity of RA evaluated by the Disease Activity Score-28 may indicate lower values than actual activities [21]. Consequently, in patients using tocilizumab, the actual disease activity may be underestimated, concealing the underlying disease activity and potentially increasing the cardiovascular risk. Given the observed association despite the limited sample size and the conflicting evidence between our findings and those of previous trials, further large-scale studies should be conducted to investigate such association in more detail. With the divergent outcomes observed in the association between tocilizumab and stroke, it is premature to definitively determine the risk. However, tocilizumab therapy should be cautiously advised for patients with RA, emphasizing the importance of raising the awareness about the potential risk of stroke in this particular population.

Sulfasalazine is a widely used csDMARD to treat RA and other inflammatory diseases, including spondyloarthritis. In a population-based retrospective cohort study, sulfasalazine at its optimal dose reduces the risk of cardiovascular diseases including stroke and AMI in patients with ankylosing spondylitis (HR: 0.65) [22]. Another case-control study showed a significant decrease in cardiovascular risk associated with sulfasalazine exposure (OR: 0.37, 95% CI: 0.14–0.99) in patients with RA compared with those who were not exposed to sulfasalazine [23]. This decrease in sulfasalazine-related cardiovascular risk may be attributed to the anti-inflammatory effects of the drug and its inhibition of arachidonic acid-mediated platelet aggregation [24]. Similarly, our study found that current long-term exposure to sulfasalazine reduced the stroke risk (aOR: 0.75, 95% CI: 0.59–0.94) in patients with RA.

Hydroxychloroquine reduces the risk of atherosclerosis by affecting lipid, glucose, coagulation, and endothelial function in patients with RA and systemic lupus erythematosus [25,26]. However, concerns about the increased risk of heart failure and arrhythmia associated with hydroxychloroquine have been raised [27]. In a recent study using Medicare data, the risk of MACE associated with hydroxychloroquine in patients with a history of heart failure is higher than that related to methotrexate [28]. However, in another retrospective study involving over 140,000 hydroxychloroquine-treated patients with RA, the incidence of ischemic stroke is lower (HR: 0.824) [25]. Similarly, the present study showed that current exposure to hydroxychloroquine was significantly associated with a decreased risk of stroke.

Unlike bDMARDs, JAK inhibitors have been relatively recently introduced for RA treatment. The Oral Rheumatoid Arthritis Trial (ORAL) Surveillance, a landmark post-marketing safety study, revealed an association between tofacitinib and a higher risk of MACE compared to anti-TNF-α agents in patients with RA aged ≥50 years with cardiovascular risk factors, thereby raising concerns [29]. However, subsequent studies reported more nuanced results regarding the cardiovascular safety of JAK inhibitors. In particular, a nationwide population-based cohort study showed that the risk of MACE, including ischemic stroke, did not significantly differ between JAK inhibitors and adalimumab, even in patients with high cardiovascular risk [30]. Similarly, another cohort study reported a comparable risk of MACE between patients treated with JAK inhibitors and those treated with IL-6 receptor inhibitors [31]. In our study, only 9 (0.7%) patients used JAK inhibitors before they had a stroke event, thereby limiting our ability to draw definitive conclusions about the relationship between JAK inhibitors and stroke risk. Further studies should involve a larger number of patients using JAK inhibitors.

The present study had some limitations. First, the HIRA claims database lacks detailed clinical data on lifestyle factors (smoking, alcohol consumption, physical activity, and diet), medication adherence, and detailed regimens, including tapering of glucocorticoid doses (limited to the total prescribed amount); these unmeasured confounders could potentially bias our findings. We also could not stratify the severity of diabetes by insulin use for risk adjustment. Second, the occurrence of a stroke was identified on the basis of the registered diagnostic and imaging codes, which might not fully capture the severity of the stroke. Third, both channeling bias and reverse causation might have influenced our results, as patients with severe RA were more likely to initiate treatment with glucocorticoids, bDMARDs (including tocilizumab), or JAK inhibitors, and these medications might have been preferentially prescribed to patients with a higher baseline risk of stroke (for instance, due to uncontrolled inflammation or comorbidities), potentially reflecting selection bias where treatment allocation is influenced by disease severity or underlying risk factors. Fourth, we were unable to adjust for RA disease activity owing to a lack of clinical measures, such as Disease Activity Score-28, CRP levels, and erythrocyte sedimentation rate, in the claims database, which represents a major limitation. While proxy indicators such as bDMARDs use patterns and CCI were used, these cannot fully capture RA disease activity. bDMARDs use may indirectly reflect disease severity, whereas the CCI primarily indicates overall health status. Consequently, the risk of stroke associated with medications might reflect differences in disease severity rather than direct drug effects. Additionally, patients prescribed hydroxychloroquine or sulfasalazine typically exhibit lower disease activity than those requiring bDMARDs or JAK inhibitors, leading to an inherent underestimation of the risk of stroke in these groups due to confounding by indication. Finally, the small number of patients treated with specific medications affected our statistical estimates. The limited sample size precluded subtype analysis for bDMARDs (particularly tocilizumab with only 16 cases of current exposure) and JAK inhibitors (recently introduced into clinical practice) despite conflicting findings regarding cardiovascular risk in recent studies such as the ORAL Surveillance. The lack of multiple-comparison adjustment, observed reverse exposure–time relationship with tocilizumab, and discrepancies between our findings and the results of randomized controlled trials necessitate cautious interpretation of the results. Nevertheless, the major strength of this study lies in its real-world design that utilized a nationwide claims database involving a large population to assess the effects of various anti-rheumatic agents on the risk of stroke. To our knowledge, this research is the first nested case-control cohort study focusing on the association between the risk of ischemic stroke in RA and the duration and timing of various anti-rheumatic agents.

In conclusion, this study showed that current exposure to sulfasalazine and hydroxychloroquine was associated with a decreased risk of stroke, while current exposure to glucocorticoid and tocilizumab was related to an increased risk of incident stroke. The associations between tocilizumab use and incident stroke should be cautiously interpreted due to the small number of tocilizumab users, unmeasured confounders, and the reversed exposure-time relationship. Therefore, clinicians should be aware of the elevated risk of stroke in patients with RA and carefully consider the risk of ischemic stroke when selecting anti-rheumatic agents.

## Supporting information

S1 Table Operational definitions of inclusion/exclusion criteria. (DOCX)

S2 Table Operational definitions of stroke. (DOCX)

S3 Table Comparison between medication exposure at any time prior to the index date and current exposure (within 3 months) in cases and controls. (DOCX)
