# Supplementary material for: Risk of ischemic stroke associated with anti-rheumatic agents in patients with rheumatoid arthritis: A nationwide population-based case-control study
Source: PLoS One. 2025 Jun 17;20(6):e0326311. doi: 10.1371/journal.pone.0326311 (PMC12173416; doi:10.1371/journal.pone.0326311)
Supplement: S1 Table — (DOCX) [file pone.0326311.s001.docx]

**S1 Table.** **Operational definitions of inclusion/exclusion criteria.**

| **Category** | **Disease** | **ICD-10 codes (RID code)** |
| --- | --- | --- |
| Inclusion | Rheumatoid arthritis | M05 (V223) |
| Exclusion | Acute myocardial infarction | I21 |
|  | Systemic lupus erythematosus | M32 |
|  | Mixed connective tissue diseases | M351 |
|  | Sjogren’s syndrome | M350 |
|  | Inflammatory myositis | M331, M332 |
|  | Interstitial lung diseases | J84 |
|  | Solid organ transplantation | Z940, Z941, Z942, Z943, Z944, and Z948 |
|  | Cancer | C00-97 and cancer registration codes (V027, V193, and V194) |
|  | HIV infection | B20-24, R75, Z21 |
|  | Dialysis | National Health Insurance Payment codes (O701x, O702x,  O703x, O704x, O705x, O706x, O707x, and O708x) |
| Comorbidities | Hypertension | I10-13, I15 |
|  | Dyslipidaemia | E78 |
|  | Diabetes mellitus | E10-14 |
|  | Carotid stenosis | I652 |
|  | Asthma | J45-46 |
|  | Chronic obstructive pulmonary disease | J44 |
|  | Chronic kidney disease | N18-19 |
|  | Congestive heart failure | I50 |
|  | Atrial fibrillation | I48 |

Abbreviations: ICD-10, Tenth revision of International Statistical Classification of Diseases and Related Health Problems; RID, rare intractable disease.
