# Supplementary material for: Risk of ischemic stroke associated with anti-rheumatic agents in patients with rheumatoid arthritis: A nationwide population-based case-control study
Source: PLoS One. 2025 Jun 17;20(6):e0326311. doi: 10.1371/journal.pone.0326311 (PMC12173416; doi:10.1371/journal.pone.0326311)
Supplement: S2 Table — (DOCX) [file pone.0326311.s002.docx]

**S2 Table. Operational definitions of stroke.**

| **Event** | **Criteria** | **Code or definition** |
| --- | --- | --- |
| Acute ischemic stroke | Diagnosis | Cerebral infarction (I63) |
|  | Code related to stroke procedure or brain imaging | Brain MRI (HE101, HE201, HE501), hippocampus MRI (HE102, HE202, HE502), brain MRA (HE135, HE235, HE535, HI135, HJ135, HI235,HJ235, HI535, HJ535), neck MRA (HE136, HE236, HE536, HI136, HJ136, HI236, HJ236, HI536, HJ536), MRI-diffusion (HF101, HF201), MRI-perfusion (HF102, HF202), MRI-spectroscopy (HF103, HF203), MRI-cine (HF104), MRI-dynamic (HF105, HF305), MRI-dual-contrast (HF106, HF306), MRI-functional (HF107), limited CT (HA441), brain CT (HA451 , HA461 , HA471) , vertebral angiography (HA601), common carotid angiography (HA602), external carotid angiography (HA603), internal carotid angiography (HA604, HA606), vessel angiography (HA605), Percutaneous Trans-luminal Angioplasty (M6593), Percutaneous Intra-vascular Installation of Metallic Stent (M6601), Thrombolysis : Intracranial vessels (M6630) |
|  | Type of visit | Claims related to admission |

Abbreviations: CT, computed tomography; MRA, magnetic resonance angiography; MRI, magnetic resonance imaging.
