# Supplementary material for: Risk of ischemic stroke associated with anti-rheumatic agents in patients with rheumatoid arthritis: A nationwide population-based case-control study
Source: PLoS One. 2025 Jun 17;20(6):e0326311. doi: 10.1371/journal.pone.0326311 (PMC12173416; doi:10.1371/journal.pone.0326311)
Supplement: S3 Table — (DOCX) [file pone.0326311.s003.docx]

**S3 Table. Comparison between medication exposure at any time prior to the index date and current exposure (within 3 months) in cases and controls.**

|  | Exposure at any time  (prescription any time prior to the index date) | | Current exposure  (prescription within 3 months of the index date) from Table 2 | |
| --- | --- | --- | --- | --- |
| **Medication** | Case | Control | Case | Control |
|  | (n=1,384) | (n=5,499) | (n=1,384) | (n=5,499) |
| Hydroxychloroquine | 703 (50.8) | 2918 (53.1) | 433 (31.3) | 1799 (32.7) |
| Methotrexate | 1045 (75.5) | 4167 (75.8) | 795 (57.4) | 3042 (55.3) |
| Leﬂunomide | 563 (40.7) | 1885 (34.3) | 436 (31.5) | 1315 (23.9) |
| Sulfasalazine | 287 (20.7) | 1270 (23.1) | 159 (11.5) | 749 (13.6) |
| Tacrolimus | 164 (11.8) | 530 (9.6) | 109 (7.9) | 339 (6.2) |
| Glucocorticoids | 1180 (85.3) | 4289 (78.0) | 1055 (76.2) | 3308 (60.2) |
| Anti-TNF-α agents | 78 (5.6) | 280 (5.1) | 35 (2.5) | 173 (3.1) |
| Abatacept | 13 (0.9) | 34 (0.6) | 6 (0.4) | 26 (0.5) |
| Tocilizumab | 20 (1.4) | 39 (0.7) | 16 (1.2) | 21 (0.4) |
| Janus kinase inhibitors | 8 (0.6) | 17 (0.3) | 7 (0.5) | 13 (0.2) |

Abbreviations: TNF: tumor necrosis factor
